# Comparison of breath‐hold and free‐breathing positions of an external fiducial by analysis of respiratory traces

**DOI:** 10.1120/jacmp.v9i3.2768

**Published:** 2008-06-23

**Authors:** Sandeep Hunjan, George Starkschall, Isaac Rosen, Karl Prado, Naresh Tolani, Peter Balter

**Affiliations:** ^1^ Department of Radiation Physics The University of Texas M. D. Anderson Cancer Center Houston TX USA

**Keywords:** respiratory monitoring, ITV, breath‐hold, lung tumor

## Abstract

An internal target volume (ITV) accounting for respiratory‐induced tumor motion is best obtained using 4DCT. However, when 4DCT is not available, inspiratory/expiratory breath‐hold $\left({\text{BH}}_{\text{insp}},\;{\text{BH}}_{\text{exp}}\right)$ CT images have been suggested as an alternative. In such cases, an external fiducial on the abdomen can be used as a substitute for tumor motion and CT images are acquired when the marker position matches – as judged by the therapist/physicist ‐ its positions at previously determined free‐breathing (FB) respiratory extrema $\left({\text{FB}}_{\text{insp}},\;{\text{FB}}_{\text{exp}}\right)$. In this study we retrospectively determined the accuracy of these matches. Free breathing 4DCT images were acquired, followed by ${\text{BH}}_{\text{insp}}$ and ${\text{BH}}_{\text{exp}}$ CT images for 25 patients with non‐small‐cell lung cancer. Respiration was monitored using a commercial external fiducial system, which generates positional information while CT studies are conducted. Software was written for statistically analyzing the displacement of the external fiducial during ${\text{BH}}_{\text{insp}}$ and ${\text{BH}}_{\text{exp}}$ CT acquisition and comparing these displacements with corresponding mean FB extrema positions $\left({\text{FB}}_{\text{insp}}\;\text{and}\;{\text{FB}}_{\text{exp}},\;\text{respectively}\right)$ using a Student's *t*‐test. In 72% of patients, mean positions at ${\text{BH}}_{\text{insp}}$ differed significantly from mean positions at ${\text{FB}}_{\text{insp}}$ (p<0.05: 0.13–1.40 cm). In 92% of patients, mean positions at ${\text{BH}}_{\text{exp}}$ differed significantly from mean positions at ${\text{FB}}_{\text{exp}}$ (p<0.05: 0.03–0.70 cm), although this difference was smaller than 0.5 cm in many cases (median=0.34 cm). Our findings indicate that relying solely on abdominal external markers for accurate BH CT imaging in order to accurately estimate FB extrema positions may be subject to significant error.

PACS numbers 87.53.bd, 87.57.C‐, 87.59.Fm, 87.55.Gh

## I. INTRODUCTION

Because of respiratory motion, lung tumors may move up to 2 cm during a single fraction during radiation therapy.^1^, ^4^ To account for respiratory motion in designing radiation therapy plans, the International Commission on Radiation Units and Measurements (ICRU) has identified the internal target volume (ITV) as encompassing the entire range of motion of a tumor, both demonstrably and microscopically, during treatment delivery.^(5)^ Initially, researchers defined population‐based margins for expanding the clinical target volume to generate the ITV. State‐of‐the‐art radiation therapy simulation can define the ITV based on the extent of tumor motion explicitly measured using four‐dimensional computed tomography (4DCT).^(6)^ Consequently, margins that account for respiratory motion can now be made patient‐specific, in both magnitude and direction, resulting in better coverage for tumors with a great deal of motion and a significant decrease in the amount of irradiated uninvolved lung for tumors with little motion.^(7)^

The technology that enables explicit ITV determination based on 4DCT requires a high‐end multislice CT scanner, as well as 4DCT reconstruction software. This exclusive combination of hardware and software may be prohibitively expensive for smaller institutions. Furthermore, because 4DCT images are taken while the patient is freely breathing, they can still contain residual motion blurring and artifacts. Therefore, an alternative procedure that has been suggested to determine the ITV is breath‐hold (BH) CT imaging, in which the patient holds his or her breath during imaging at both end‐inspiration $\left({\text{BH}}_{\text{insp}}\right)$ and end‐expiration $\left({\text{BH}}_{\text{exp}}\right)$.^(8)^

The suitability of BH imaging for ITV determination in patients who undergo treatment while freely breathing is unknown. Even if one accounts for hysteresis and the non‐rectilinear tumor motion between extrema positions,^(9)^ how well the tumor positions at BH respiratory extrema reflect the tumor positions at free‐breathing (FB) respiratory extrema remains unclear. Moreover, a common method of monitoring the respiratory cycle is the use of external fiducials placed on the abdomen, whose correlation with internal tumor positions is still questionable.^(10)^ Yet it is assumed during BH imaging that the positions of the external fiducial matches its corresponding positions at FB respiratory extrema. The simplest approach to determine appropriate BH positions is to rely on the patient's estimation of FB extrema positions with no external marker. However, this method results in no quantitative measurement of the BH position accuracy.

We performed the present retrospective study to quantitatively determine how accurately BH positions – as defined using an external marker on the abdomen ‐ reflected free‐breathing (FB) respiratory extrema positions in patients who had routinely undergone BH CT imaging for ITV construction. During institutional conversion from multiple BH CT studies to FB 4DCT for lung tumor ITV‐generation, we investigated the suitability of FB 4DCT for deriving tumor ITVs. During this period, combined FB 4DCT and BH scans were performed at our institute under an Institutional Review Board (IRB) approved protocol. Although this combined, redundant study would not be routinely performed by institutions ‐ and is no longer done at our own institute ‐ the scans from this investigative period provided us with the opportunity for this retrospective study. Also, although the use an external fiducial system might be economically unfeasible for a small institution without 4DCT capabilities, we were in the position to exploit the Varian RPM system for quantitative assessment of BH position in this study.

## II. METHODS AND MATERIALS

The study population consisted of 25 patients who underwent radiation therapy for non‐small‐cell lung cancer from December 1, 2004 to January 19, 2005, at The University of Texas M. D. Anderson Cancer Center. During this period, free‐breathing (FB) 4DCT and breath‐hold (BH) CT scans were routinely performed for treatment‐planning purposes. The patient data were retrospectively acquired under an IRB‐approved retrospective chart‐review protocol.

Patients underwent scanning using one of two multislice helical CT scanners: Mx8000 IDT (Philips Medical Systems, Cleveland, OH) and Discovery ST (GE Healthcare, Waukesha, WI). Immobilzation was achieved in the standard supine position using a wing board with a T‐bar handgrip in conjunction with a vacuum immobilization device (BlueBAG Vacuum Cushions; Medical Intelligence, Schwabmünchen, Germany).

Real‐time monitoring of patient respiration during FB 4DCT and BH CT scanning was accomplished using an external fiducial device (RPM; Varian Medical Systems, Palo Alto, CA) that measured the vertical displacement of the abdomen during respiration. The position of a reflector on the surface of a block positioned on each patient's abdomen was tracked using an infrared light source and a charge‐coupled device (CCD) detector. Patients were allowed to observe their respiratory motion by viewing a liquid crystal display (LCD) flat‐panel video monitor (Optiview, Rancho Dominguez, CA) mounted vertically at the end of the treatment couch through a mirror assembly.^(11)^ Some of the patients later used virtual reality goggles (i‐O Display Systems, Sacramento, CA) rather than the monitor/mirror assembly for visual feedback, as the latter interfered less with patient setup and immobilization. Neither visual feedback method was deemed to be more accurate or superior to the other. The choice of visual feedback method depended on patient preference. Some patients could not tolerate wearing goggles, whereas the positioning of others did not allow the use of the monitor/mirror assembly method. Either way, by having a method to observe their own breathing traces, patients were able to exert finer control over the position of breath‐hold.

Prior to acquiring the CT images during breath hold, the patients' respiration was monitored to estimate the average FB amplitude of fiducial motion during normal respiration. For BH imaging, patients were shown a horizontal bar that moved vertically, tracking the motion of the fiducials in real‐time within a color‐coded region that corresponded to the previously determined average FB amplitude. The patients thus obtained visual feedback that displayed the real‐time position of the fiducial during CT image acquisition as well as target positions for their breath‐holds corresponding to the upper and lower limits of the color‐coded region. Patients were instructed to observe a trace of their respiratory cycle and perform a BH at end‐inspiration $\left({\text{BH}}_{\text{insp}}\right)$ and end‐expiration $\left({\text{BH}}_{\text{exp}}\right)$, corresponding to the upper and lower limits of the colored region.

After positioning the patient on the CT couch, the fiducial marker was placed on the patient approximately at midline and midway between the xiphoid process and umbilicus. This position usually corresponds with the maximum abdominal motion. The FB 4DCT scan was taken first, followed by the two BH scans (at end‐inspiration and end‐expiration) with no change in the patient or fiducial marker position.

Respiratory traces consisting of fiducial displacement as a function of both time and phase were obtained during the FB 4DCT and BH scans. For this study, the traces were exported as text files. A custom software program was developed to analyze these traces (MATLAB; The MathWorks, Natick, MA). This program displayed a respiratory trace on a time axis with indicators identifying the period of CT data acquisition (beam‐on). For each FB 4DCT trace, the user selected the portion of the trace corresponding to the beam‐on time, and the program automatically extracted the displacements corresponding to the end‐inspiration and end‐expiration periods of the breathing cycles within the selected time intervals. From the FB 4DCT traces, the mean position of the external fiducial at end‐inspiration $\left({\text{FB}}_{\text{insp}}\right)$ and its standard deviation $\left({\text{SD‐FB}}_{\text{insp}}\right)$ and the mean position of the external fiducial at end‐expiration $\left({\text{FB}}_{\text{exp}}\right)$ and its standard deviation $\left({\text{SD‐FB}}_{\text{exp}}\right)$ were calculated. Typically, FB statistics were extracted from the 12–15 breathing cycles required for the FB 4DCT study acquired after patients had achieved steady‐state breathing. From BH traces, the mean position of the external fiducial at end‐inspiration $\left({\text{BH}}_{\text{insp}}\right)$ and its standard deviation $\left({\text{SD‐BH}}_{\text{insp}}\right)$ and the mean position of the external fiducial at end‐expiration $\left({\text{BH}}_{\text{exp}}\right)$ and its standard deviation $\left({\text{SD‐BH}}_{\text{exp}}\right)$ were calculated.

**Figure 1:**
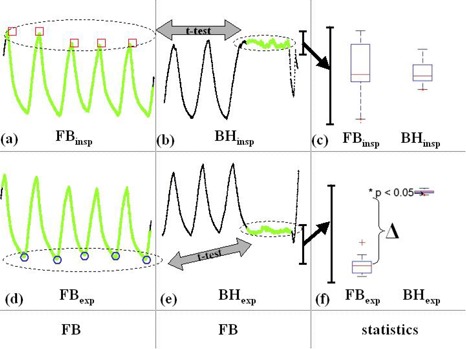
Data from Patient no. 1 summarizing the process of respiration trace analysis. Vertical direction of traces represents vertical displacement of reflective marker (cm) and the horizontal direction represents time (t) (axes omitted for clarity). Free breathing (FB) extrema positions $\left({\text{FB}}_{\text{insp}}\;\text{and}\;{\text{FB}}_{\text{exp}}\right)$ are extracted and compared to their breath‐hold (BH) counterparts (${\text{BH}}_{\text{insp}}$ and ${\text{BH}}_{\text{exp}}$, respectively) using a Student's *t*‐test. Time points at which CT data are acquired are highlighted in green. (a) End‐inspiration FB $\left({\text{FB}}_{\text{insp}}\right)$ points (red squares) are extracted from which the mean and standard deviation (SD) are calculated. (b) The mean and SD of inspiration BH $\left({\text{BH}}_{\text{insp}}\right)$ are calculated and a Student's *t*‐test was used to compare ${\text{FB}}_{\text{insp}}$ and ${\text{BH}}_{\text{insp}}$. (c) Summary of the statistical analysis (scale expanded for visual clarity) showing no significant difference between the average displacement of the two end‐inspiration datasets. The box plot has lines at the lower quartile, median, and upper quartile values. The whiskers are lines extending from each end of the box to show the extent of the rest of the data. Outliers are data with values beyond the ends of the whiskers (red crosses). A similar analysis is applied at end‐expiration. (d) Mean and SD extracted from end‐expiration FB $\left({\text{FB}}_{\text{exp}}\right)$ points (blue circles) and compared to (e) expiration BH $\left({\text{BH}}_{\text{exp}}\right)$. (f) Summary of the analysis (scale expanded for visual clarity) of end‐expiration data showing a significant difference, Δ, between the ${\text{FB}}_{\text{exp}}$ and ${\text{BH}}_{\text{exp}}$ data (p<0.05)

The differences between the displacements measured using the two methods and their uncertainty were computed for each patient. Because there was no alteration in the position of the fiducial marker between the FB 4DCT and BH scans, the positions measured during the scans were relative to the same coordinate system and could be compared directly. The differences were analyzed and tested for statistical significance using Student's *t*‐test (p ≤ 0.05 for statistical significance). Fig. 1 summarizes the process of statistical analysis of respiratory traces.

## III. RESULTS

**Figure 2:**
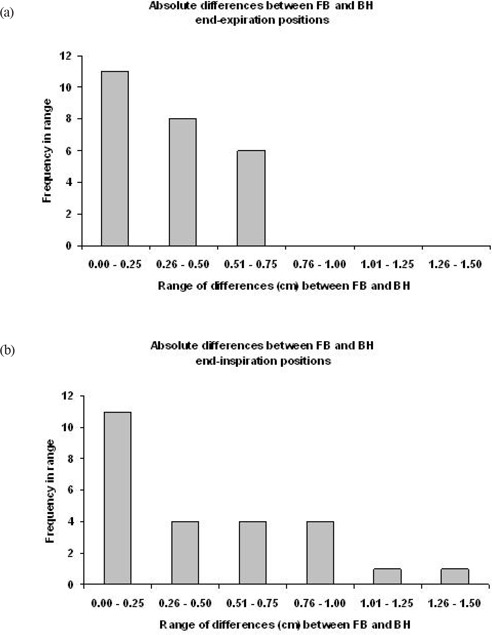
Histograms of the absolute differences between average FB and BH positions at (a) end‐expiration and (b) end‐inspiration.

Table 1 lists the absolute differences between average FB and BH positions at end‐inspiration $\left(\Delta_{\text{insp}}\right)$ and end‐expiration $\left(\Delta_{\text{exp}}\right)$, respectively. For end‐inspiration, the absolute difference between the FB and BH fiducial positions ranged from 0−1.4 cm (mean=0.4 cm, SD=0.4 cm). For end‐expiration, the absolute difference between mean FB and BH fiducial positions ranged from 0−0.7 cm (mean =0.3 cm, SD=0.2 cm). Fig. 2a represents a histogram of the absolute differences between the FB and BH end‐inspiration positions. Fig. 2b is a histogram of the absolute differences between the FB and BH end‐expiration positions. Table 2 lists the standard deviations of the fiducial positions at end‐inspiration and end‐expiration and their mean values as a measure of stability of these positions.

**Table 1:** The differences between free breathing (FB) and breath‐hold (BH) fiducial locations at inspiration and expiration positions, respectively. $\left(\Delta_{\text{insp}}=\text{magnitude of}\;({\text{FB}}_{\text{insp}}-{\text{BH}}_{\text{insp}});\;\Delta_{\text{iexp}}=\text{magnitude of}\;({\text{FB}}_{\text{exp}}-{\text{BH}}_{\text{exp}});\;\text{units in cm}\right)$.

*Patient*	$\Delta_{\text{insp}}$	$\Delta_{\text{exp}}$
1	0.04	0.66
2	0.71	0.65
3	0.23	0.03
4	0.60	0.36
5	0.76	0.30
6	0.32	0.24
7	0.02	0.06
8	0.34	0.58
9	0.91	0.51
10	0.02	0.01
11	0.39	0.49
12	0.03	0.25
13	0.94	0.60
14	0.03	0.47
15	1.40	0.70
16	0.17	0.42
17	0.98	0.03
18	0.13	0.40
19	0.49	0.06
20	0.57	0.44
21	0.01	0.19
22	1.20	0.38
23	0.66	0.04
24	0.00	0.18
25	0.09	0.09

**Table 2:** Summary of the standard deviations of mean positions of the fiducials during end inspiration and end expiration (FB – free‐breathing; BH – breath‐hold; units in cm)

	*SD insp position*		*SD exp position*	
*Patient*	*FB*	*BH*	*FB*	*BH*
1	0.17	0.07	0.06	0.01
2	0.26	0.11	0.07	0.05
3	0.21	0.03	0.09	0.03
4	0.32	0.31	0.16	0.13
5	0.07	0.04	0.07	0.04
6	0.10	0.19	0.09	0.04
7	0.11	0.05	0.04	0.02
8	0.36	0.13	0.16	0.06
9	0.25	0.08	0.08	0.01
10	0.08	0.06	0.04	0.04
11	0.10	0.04	0.07	0.41
12	0.33	0.12	0.10	0.04
13	0.39	0.31	0.09	0.07
14	0.11	0.07	0.20	0.53
15	0.17	0.14	0.16	0.06
16	0.06	0.14	0.05	0.04
17	0.31	0.05	0.41	0.06
18	0.34	0.04	0.11	0.10
19	0.15	0.09	0.09	0.09
20	0.06	0.02	0.05	0.01
21	0.15	0.11	0.06	0.12
22	0.39	0.12	0.06	0.17
23	0.13	0.06	0.03	0.02
24	0.15	0.04	0.02	0.03
25	0.47	0.06	0.22	0.05
Mean	0.21	0.10	0.10	0.09

Finally, Table 3 shows the *p* values for the differences in the FB and BH fiducial positions at inspiration (insp) and expiration (exp) extrema. In most cases, the *p* value was <0.001, indicating highly significant differences between the FB and BH extrema positions.

**Table 3:** The statistical significance of absolute differences between free‐breathing and breath‐hold extrema positions at end‐inspiration (insp) and end‐expiration (exp)

	p‐*values*	
*Patient*	*insp*	*exp*
1	0.084	<0.001
2	<0.001	<0.001
3	<0.001	<0.001
4	<0.001	<0.001
5	<0.001	<0.001
6	<0.001	<0.001
7	0.085	<0.001
8	<0.001	<0.001
9	<0.001	<0.001
10	0.232	0.214
11	<0.001	<0.001
12	0.359	<0.001
13	<0.001	<0.001
14	0.099	0.001
15	<0.001	<0.001
16	<0.001	<0.001
17	<0.001	0.401
18	<0.001	<0.001
19	<0.001	<0.001
20	<0.001	<0.001
21	0.631	<0.001
22	<0.001	<0.001
23	<0.001	<0.001
24	0.908	<0.001
25	0.005	<0.001

## IV. DISCUSSION

We performed the present study to determine how accurately BH positions – as defined using an external marker on the abdomen ‐ reflect free‐breathing (FB) respiratory extrema positions. Table 1 shows that at end‐inspiration the magnitude of the differences between the mean FB and BH fiducial positions ranged from 0−1.4 cm. In the extreme case of patient number 15, a 1.4 cm difference in fiducial positions at inspiration could result in a tumor position at BH, which is very different to that during FB at the corresponding phase of the breathing cycle. At end‐expiration, the magnitude of the differences between FB and BH fiducial positions ranged from 0−0.7 cm. These data are summarized in the histograms of Fig. 2, which show that there is less variation between BH and FB positions at end‐expiration than at end‐inspiration. Reproducibility of free‐breathing extrema positions while the patient is freely breathing, is crucial for passively‐gated respiratory‐correlated therapy. The data in Table 2 show that the average ${\text{FB}}_{\text{insp}}$ SD is greater than the average ${\text{FB}}_{\text{exp}}$ SD, suggesting that end‐expiration is the best phase for passive respiratory‐gated treatment where the patient is freely breathing, which is in agreement with previous observations.^12^, ^18^

For BH studies, we found that the mean ${\text{BH}}_{\text{insp}}$ position was significantly different (≤ 0.05: 0.13 – 1.40 cm) from the mean ${\text{FB}}_{\text{insp}}$ position in 18 (72%) patients and that the mean ${\text{BH}}_{\text{exp}}$ position was significantly different (p<0.05: 0.03–0.70 cm) from the mean ${\text{FB}}_{\text{exp}}$ position in 23 (92%) patients (Table 3). The larger number of cases which reach statistically significant difference for end‐expiration positions is due to the low variability (smaller standard deviations) of the end‐expiration FB positions as seen in Table 2. While these differences for end‐expiration positions are statistically significant, in practice the large magnitude differences are of most importance. For example, five patients had differences at end‐expiration greater than 4 mm and six additional patients had differences greater than 5 mm (Table 1). Examination of the magnitude of the difference between FB and BH fiducial positions at end‐inspiration ${|\text{FB‐BH}|}_{\text{insp}}$ versus end‐expiration ${|\text{FB‐BH}|}_{\text{exp}}$ reveals that 14 patients show larger deviation for ${|\text{FB‐BH}|}_{\text{insp}}$ than for ${|\text{FB‐BH}|}_{\text{exp}}$. Although this is a small study, these results suggest that ${\text{BH}}_{\text{exp}}$ is the more appropriate position for BH radiation therapy. Practically, however, there may be significant normal tissue sparing with the ${\text{BH}}_{\text{insp}}$ protocol as is evident during deep‐inspiration breath hold (DIBH) radiation therapy.^16^, ^19^, ^24^

One reason for the differences between the BH and FB extrema positions is that during BH scanning, patients can contract or relax their abdominal muscles, causing the external fiducial to move significantly without any corresponding changes in lung volume or tumor position. These differences are not necessarily reflective of differences in internal tumor positions during BH compared to FB extrema. However, during a typical BH CT examination the therapist qualitatively judges the integrity of patient BH solely by comparing the external fiducial BH positions to FB extrema positions. This study quantitatively addresses the accuracy of this method by statistically comparing FB and BH respiratory traces derived from an external fiducial.

Correlation between the external fiducial and internal tumor position is an important, separate topic actively being investigated in our institute as well as others. Balter et al. compared positions of tumor GTVs at FB extrema (from the 0 and 50% phases of 4DCT studies) with BH CT derived GTVs at ${\text{BH}}_{\text{insp}}$ and ${\text{Bh}}_{\text{exp}}$.^(25)^ This study showed that GTV positions during BH studies do not accurately represent the limits of the FB GTV, especially during inspiration. Similarly, with respect to the external marker, we found that the position of the fiducial during BH does not always accurately represent the limit of the fiducial position during FB. Therefore, care must be taken during BH imaging, interpretation of respiratory traces, and creation of treatment‐planning margins based solely upon external markers. Alternatively, if available, an approach other than BH CT such as multiple slow CT scans,^(26, 27)^ positron emission tomography,^(28)^ extended‐time CT, or 4DCT,^(29, 30)^ may more accurately elucidate the true extent of tumor motion during FB and provide more accurate ITVs for treatment planning.

## V. CONCLUSION

External fiducial positions during end‐inspiration end‐expiration breath‐holds do not accurately correspond to positions of the fiducial during corresponding end‐inspiration end‐expiration phases of free breathing. Our findings indicate that relying solely on abdominal external markers for accurate BH CT imaging in order to accurately estimate FB extrema positions may be subject to significant error. Consequently, an internal target volume (ITV) generated using CT scans acquired with the fiducial at these breath hold positions could be erroneous and care must be taken using such an approach. If 4DCT, which explicitly accounts for tumor motion during the breathing cycle, is not available then additional margins may be warranted in the ITV generated using surrogate breath‐hold techniques.
